# Systematic review and meta-analyses of psychosocial interventions for veterans of the military

**DOI:** 10.3402/ejpt.v3i0.19267

**Published:** 2012-12-05

**Authors:** Neil J. Kitchiner, Neil P. Roberts, David Wilcox, Jonathan I. Bisson

**Affiliations:** 1 Traumatic Stress Service, Cardiff and Vale University Health Board, University Hospital of Wales, Cardiff, UK; 2 South West Veterans Mental Health Service, The Blackberry Centre, Blackberry Hill Hospital, Fishponds, Bristol, UK; 3 School of Medicine, Cardiff University, Heath Park, Cardiff, UK

**Keywords:** Veterans, common mental disorders, randomised controlled trials

## Abstract

**Background:**

The efficacy of psychosocial therapies for common mental health disorders in veterans is unclear and requires further examination.

**Method:**

Systematic review and meta-analyses of randomised controlled trials (RCTs). Twenty databases were searched. Studies were included if they reported a psychosocial intervention designed to treat or reduce common mental health symptoms in veterans identified as being symptomatic at the time they entered the study. Studies of substance dependency disorders and psychosis were excluded. Eligible studies were assessed against methodological quality criteria and data were extracted and analysed.

**Results:**

Twenty-nine RCTs were identified. There was evidence for the use of trauma-focused therapies for post-traumatic stress disorder (PTSD) and some evidence for psychological interventions in the treatment of borderline personality disorder, depression, insomnia, and panic disorder co-morbid to PTSD. However, methodological quality of many of the studies was less than optimal.

**Conclusions:**

Trauma-focused psychological therapies are likely to be effective for combat-related PTSD but there is a need for more research to determine the efficacy of psychological treatments for other mental health disorders in veterans.

Military personnel are considered one of the highest risk occupational groups for exposure to traumatic and adverse events (Hoge et al., 2002). Such events and the demands of being deployed away from family and social support increase vulnerability to a range of mental health problems, in particular to various anxiety and depressive disorders (Browne et al., 2007; Iversen, Waterdrinker, et al., 2007; King's Centre for Military Health Research [KCMHR], 2010; Prigerson, Maciejewski, & Rosenheck, 2001).

Engaging veterans (ex-service personnel who have left the military) into mental health treatment programmes remains challenging due to a variety of factors, including stigma, perceived weakness in acknowledging emotional difficulties, and the military macho culture (KCMHR, 2010). This is compounded by limited evidence to determine what treatment programmes are efficacious, resulting in a lack of consensus as to what should be offered (Creamer, Forbes, Biddle, & Elliott, 2002; Richardson, Naifeh, & Elhai, 2007). More than 60% of US Iraq veterans who screened positive for a mental health problem did not seek treatment (Hoge, 2004). Only 23% of serving UK personnel with common mental health problems were receiving medical professional help, mainly in primary care (Iversen et al., 2010).

A number of psychosocial treatments for mental health problems have been shown to be effective in civilian populations, including cognitive, behavioural, interpersonal, and mindfulness therapies (de Mello, de Jesus Mari, Bacaltchuk, Verdeli, & Neugebauer, 2005; Ehlers & Clark, 2000; Ghosh & Marks, 1987; Hofmann, Sawyer, Witt, & Oh, 2010). It is unclear whether these same treatments have similar efficacy in military veteran populations or how well military veterans engage with them (Beidel, Frueh, Uhde, Wong, & Mentrikoski, 2011).

The Institute of Medicine (IOM) concluded that the evidence is still “inadequate to address the specific interventions, settings, and lengths of treatment that are applicable in the veteran population” (Institute of Medicine Committee, 2007). Studies have shown that veterans with complex psychiatric problems are often difficult to treat with less positive outcomes than non-veterans. For example, in a meta-analysis of 26 post-traumatic stress disorder (PTSD) trials, only five included combat veterans and the overall effect size for veterans was significantly lower for other groups (Bradley, Greene, Russ, Dutra, & Westen, 2005; Chemtob, Novaco, Hamada, & Gross, 1997; Foa, Keane, Friedman, & Cohen, 2009; Glynn et al., 1999). There may be unique aspects to conditions such as PTSD in veterans (Institute of Medicine Committee, 2007).

Early dysfunctional relationships and poor attachments have been associated with mental health problems following exposure to combat and may influence poor outcome in treatment (Iversen, Fear, et al., 2007; LeardMann, Smith, & Ryan, 2010). The degree of exposure to trauma, experience of continuous threat, serving unit ethos, and moral injury may also be factors that influence psychopathology in veterans (Litz et al., 2009). In order to examine the efficacy of psychosocial interventions for veterans in reducing common mental health disorders, a comprehensive systematic review, and meta-analyses of randomised controlled trials (RCTs) was performed to evaluate the efficacy and relative effectiveness of psychosocial treatments.

## Method

A systematic bibliographic search was undertaken to locate and retrieve RCTs of psychosocial treatments for common mental health disorders from 20 databases (including EMBASE, Medline, PsycINFO, PILOTS, CINAHL, and the Cochrane Library). The key themes devised for the search strategy included veterans and military, mental health, psychotherapies, reviews and systematic reviews, psychosocial or care pathway. Each of the 20 databases was searched from inception to January 2012. The search was restricted to papers with English language abstracts. Additional published, unpublished, and in-press studies were found by hand-searching (reviewing) the references of retrieved articles, previous systematic reviews, and meta-analyses of psychosocial treatments for common mental health disorders (Bisson et al., 2007), the proceedings of meetings of both the European and International Society of Traumatic Stress Studies between 2006 and 2010 and by contacting a number of international experts within the veteran mental health field to attempt to identify unpublished studies.

### Selection

Studies were considered if they reported a psychosocial intervention designed to treat or reduce common mental health symptoms, for example, anxiety or depressive disorders in veterans who were identified as being symptomatic at the time they entered the study. The authors excluded studies of substance misuse and psychosis in order to focus on common mental health disorders within veterans. For the purpose of this review, a psychosocial intervention was defined as:

any specific non-pharmaceutical intervention aimed at reducing a range of symptoms, offered by one or more health professional or lay person, with contact between therapist and participant on at least one occasion.

It was decided *a priori* to include all forms of psychosocial therapy. Studies had to be of randomised controlled design, with adult (>16 years old) participants who had previously served in the armed forces regardless of gender, age, and country of origin. The studies had to report at least pre- and post-treatment outcomes and retain at least 50% of the original sample at the post-treatment assessment. The first author undertook the searching. Decisions as to whether individual studies met the inclusion criteria were made independently by three authors (NJK, NPR, and DW). Any disagreements were resolved by discussion between the three reviewers. When consensus could not be achieved, advice was sought from the fourth author (JIB).

### Study characteristics

An initial narrative synthesis was undertaken to describe the scope (participants, settings, intervention type, comparators, and measures of effect), quality, and outcomes of the studies. Three main efficacy outcomes were considered: retaining a diagnosis of a common mental health disorder, assessor-rated, and self-reported symptom severity via validated self-report instruments. We decided *a priori* that our primary outcomes would be clinician-rated symptom severity, although in practice this was not present for many studies.

### Quantitative data synthesis

#### Validity assessment rating tool

All published and unpublished papers were assessed against the following quality criteria as described by the Cochrane Collaboration: sequence generation, allocation concealment, blinding of assessors, exclusion criteria and drop out, and completeness of outcome data (Higgins & Green, 2009).

#### Data extraction

Study details including the disorder of interest, participants’ characteristics and type of intervention were entered into Review Manager version five (The Cochrane Collaboration, 2008). The quality criteria and accuracy of outcome data were evaluated independently by three reviewers; disagreement was handled by the same method as disagreements about selection.

Quantitative data were used to synthesise post-treatment and follow-up data where appropriate. None of the included studies provided dichotomous data for further analysis. Post-treatment data (or change scores if reported instead) for the psychosocial treatment and control condition were entered in Review Manager tables for analysis through standardised mean difference (SMD). When intention to treat (ITT) data was available, this was reported in the results. Attempts were made to access ITT data by contacting the corresponding author for a complete data set. Reasons given by authors for not being able to provide complete data included the study being old and individuals had changed employment or retired. Completer-only analysis was reported when this was the only data source available.

### Heterogeneity

A visual inspection of the forest plots was initially used to explore for possible heterogeneity. It was also measured by observing the I^2^ test (used to measure of the consistency between trials in a meta-analysis) (Higgins & Green, 2009). An *a priori* decision was made to use a random-effects model when the I^2^ was 30% or greater. However, because of the degree of clinical heterogeneity of studies included, a post hoc decision was taken to use a random effects model in all analyses undertaken.

### Study characteristics

Tables 1 and 2 provide details of the authors, method, participants, interventions, and clinical measures, length of follow-up and outcome of the 29 studies included. A total of 28 originated from the United States and one from Australia (Devilly, Spence, & Rapee, 1998). Participants in 16 studies were male Korean/Vietnam veterans with combat-related chronic PTSD (Beidel et al., 2011; Bormann, Thorp, Wetherell, & Golshan, 2008; Carlson, Chemtob, Rusnak, Hedlund, & Muraoka, 1998; Chemtob et al., 1997; Cook et al., 2010; Devilly et al., 1998; Dunn et al., 2007; Frueh et al., 2007; Glynn et al., 1999; Keane, Fairbank, Caddell, & Zimering, 1989; Monson et al., 2006; Morland et al., 2010; Morland, Pierce, & Wong, 2004; Schnurr et al., 2003; Teng et al., 2008; Watson, Tuorila, Vickers, Gearhart, & Mendez, 1997). The remaining mental health disorders examined included borderline personality disorder, depression, Gulf War illness, panic disorder, and insomnia.

**Table 1:** Studies included in meta-analysis and quality outcome ratings

Authors	Participants	Interventions	Sequence generation	Allocation concealment	Blinding of participants, personnel and outcome	Exclusion criteria and refusals number reported	Incomplete outcome data	Key outcomes as reported by study authors
Depression								
Dobscha et al. (2006)	41 primary care clinicians and 375 veterans with depression.	Clinicians received depression education, then randomly assigned to 12 months of depression decision support vs. usual care.	Adequately described	Adequately described	Adequately described	Adequately described	Adequately described	Post intervention depression scores improved in both groups and differences were not significant (*N*=316, SMD=0.01, 95% CI −0.14, 0.16). The intervention group reported greater satisfaction (*p*=0.002) and were more likely to have had at least one mental health specialty appointment (41.1% vs. 27.2%; *p*=0.025), to have received an anti-depressant (79.3% vs. 69.3%; *p*=0.041) and to have received antidepressants for 90 days or more (76.2% vs. 61.6%; *p*=0.008).
Fortney et al. (2007)	395 elderly predominately male veterans with physical and behavioural health problems. From VA community-based outpatient clinics.	Participants received either usual care (*N*=218) vs. Collaborative care via telepsychiatry (*N*=177) for 12 months.	Potential bias	Potential bias	Adequately described	Potential bias	Adequately described	Participants within the experimental arm (collaborative care via telemedicine) were more likely to be adherent at both 6 months (OR=2.1, *p*=0.04) and 12 months (OR=2.7, *p*=0.01). Intervention patients were more likely to respond by 6 months (OR=2.0, *p*=0.02) and remit by 12 months (OR=2.4, *p*=0.02). Intervention patients reported larger gains in mental health status and health-related quality of life, and reported higher levels of satisfaction.
Hedrick et al. (2003)	354 veterans with major depression and/or dysthymia.	Participants received either collaborative care (*N*=168) vs. consult-liaison (*N*=186) for a period of 9 months.	Potential bias	Potential bias	Adequately described	Adequately described	Adequately described	Collaborative care produced greater improvement compared to consult-liaison in depressive symptoms from baseline to 3 months though this was not significant (*N*=354, SMD= −0.07, 95% CI−0.21, 0.07). At 9 months follow-up there was no significant difference.
Oslin et al. (2003)	97 Vietnam veterans with depression and/or at risk drinking.	Participants received either telephone disease management (TDM) treatment (*N*=46) or usual care (*N*=51).	Potential bias	Potential bias	Potential bias	Adequately described	Potential bias	Results favoured participants referred to TDM compared with those assigned to usual care (39.1% responded vs. 17.6% *p*=0.022). (WALD=5.27; 1 df; OR=0.33) (95% CI 0.13, 0.85, *p*=0.022).
Ross et al. (2008)	223 veterans within VA primary care setting with minor depression or distress.	Participants received either usual care (*N*=93) or close monitoring via telephone (*N*=130).	Potential bias	Potential bias	Potential bias	Potential bias	Potential bias	Participants in the CM exhibited fewer psychiatric diagnosis (χ^2^=4.04, 1 df, *p=*0.004), and improved overall physical health (SF-12, *M*=45.1, SD=11.8 vs. *M*=41.5, SD=12.4) (χ^2^=5.90, 1 df, *p*=0.02).
Insomnia								
Edinger and Sampson (2003)	20 veterans attending a VA medical centre with chronic primary insomnia.	Two sessions of abbreviated CBT (*N*=10) vs. sleep hygiene control (*N*=10).	Potential bias	Potential bias	Potential bias	Adequately described	Potential bias	ACBT demonstrated significant improvement in most of the outcome measures than SHC. Approximately 52% of ABCT participants reported at least 50% reduction in their wake time after sleep onset, with 55.6% of ACBT achieved normal ISQ scores at the 3 month FU (*p*=0.45).
Edinger et al. (2009)	81 Vietnam veterans with Insomnia. Within the co-morbid insomnia group most participants suffered with co-morbid depression or combat-related PTSD.	Four bi-weekly sessions CBT (*N*=40) vs. sleep-hygiene educational control therapy (*N*=41).	Potential bias	Potential bias	Adequately described	Adequately described	Potential bias	The CBT intervention demonstrated more improvement across several outcome measures in patients with both primary and co-morbid insomnia. There were significant reductions in the CBT group in insomnia symptoms (*p*=0.02) compared to sleep-hygiene participants. As well as significant reductions in unhelpful beliefs about sleep (*p*=0.04).
Nakamura et al. (2010)	63 male and female veterans with sleep disturbance (aged 18–70) and co-morbid symptoms.	Two sessions, once weekly of either sleep hygiene (SH) (*N*=28) vs. mind-body bridging (MBB) (*N*=35).	Adequately described	Adequately described	Potential bias	Adequately described	Potential bias	Sleep disturbance deceased in both groups post intervention, but was significantly better in MBB (*p*=0.028) and effect size of .74. Improvement in sleep for MBB was greater at post (28.0, ES=1.89) vs. SH (14.8, ES=0.71); *p*=0.012).
Post-traumatic stress disorder								
Carlson et al. (1998)	35 Vietnam (except one) combat veterans with PTSD.	Twelve 2 weekly individual 60–75 min sessions of EMDR vs. biofeedback assisted relaxation vs. routine clinical care.	Potential bias	Potential bias	Potential bias	Potential bias	Potential bias	Substantial decreases from pre-treatment to post-treatment on self-report PTSD severity depression and CAPS total frequency. Total number (*N*=22, SMD= −0.91, random effects, CI 95% [−1.80, −0.02].
Devilly et al. (1998)	51 Australian Vietnam combat veterans with PTSD.	Two 90 min weekly sessions of EMDR vs. equivalent without EMDR vs. standard psychiatric support.	Potential bias	Potential bias	Adequately described	Potential bias	Potential bias	No difference was observed between the groups post treatment or at 6 months follow-up (*N*=22, SMD= −0.03, 95% CI −0.87, 0.81).
Keane et al. (1989)	24 Vietnam combat veterans with PTSD.	Fourteen weekly sessions of implosive flooding vs. waiting list.	Potential bias	Potential bias	Potential bias	Potential bias	Potential bias	The intervention group demonstrated significant improvement in re-experiencing symptoms of PTSD (*N*=24, SMD= −0.22, 95% CI −1.03, 0.58), anxiety and depression, but not in numbing and social avoidance related to PTSD.
Monson et al. (2006)	60 (80%) Vietnam combat veterans with PTSD.	Twelve twice weekly sessions of cognitive processing therapy (CPT) vs. waiting list.	Potential bias	Adequately described	Potential bias	Adequately described	Adequately described	There were significant improvements in PTSD and co-morbid disorders within the CPT group compared to the wait list control (*N*=60, SMD= −23.89, 95% CI −25.81, −21.97). Forty percent (*N*=12) of the CPT group and 3% (*N*=1) of WL no longer met criteria for a PTSD diagnosis and 50% had a reliable change in PTSD symptoms at post-treatment assessment.

Other theatres included the Gulf War 1990/1991 (Donta et al., 2003), two studies contained female veterans who had never deployed (Koons et al., 2001; Price, McBride, Hyerle, & Kivlahan, 2007) and the remaining studies did not collect or report this data. The number of veterans randomised to the trials ranged from 14 (Price et al., 2007) to 395 (Fortney et al., 2007). Eleven studies included sample sizes of over 100 individuals (Cook et al., 2010; Dobscha et al., 2006; Donta et al., 2003; Dunn et al., 2007; Fortney et al., 2007; Hedrick et al., 2003; Morland et al., 2010; Ross, TenHave, Eakin, Difilippo, & Oslin, 2008; Ruskin et al., 2004; Schnurr et al., 2003; Schnurr, Friedman, Engel, Foa, & Shea, 2007). There were three studies with less than 20 participants (Chemtob et al., 1997; Morland et al., 2004; Price et al., 2007).

## Results

**Fig. 1:**
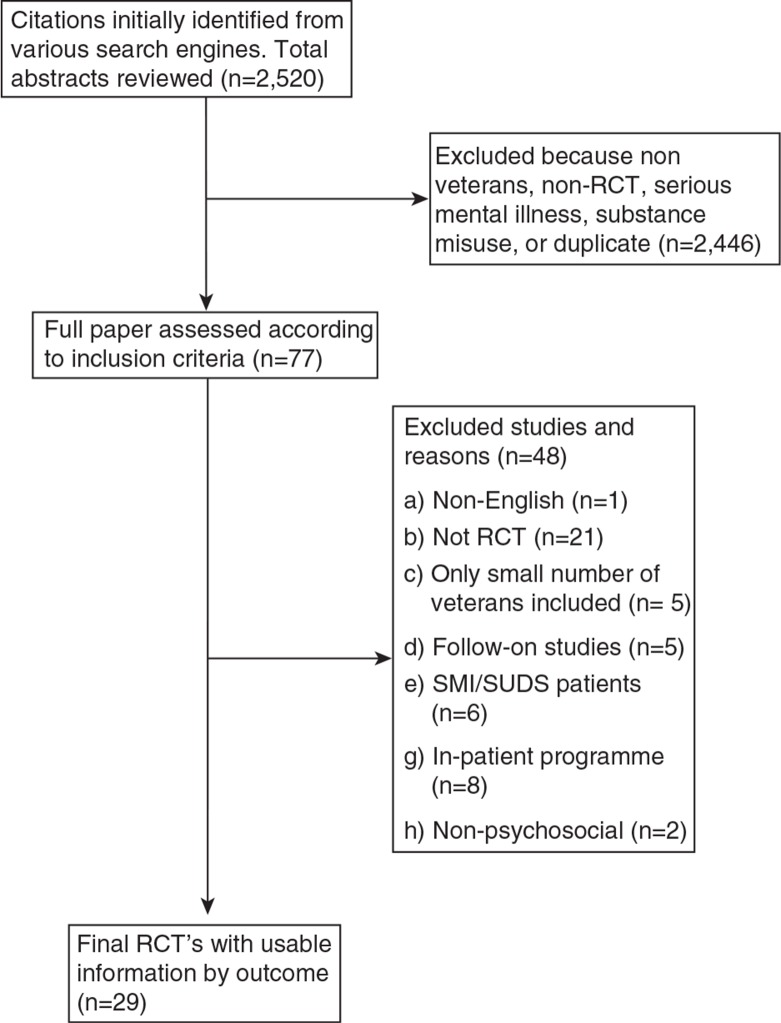
profile summarising trial flow.

Figure 1 shows the systematic review profile summarising trial flow. Of the 29 RCTs, only 12 could be used within the meta-analyses due to lack of available data or a similar comparison study, and will be presented here. The remaining studies are described in Table 2.

**Table 2:** Studies not included in meta-analysis and quality outcome ratings

Authors	Participants	Interventions	Sequence generation	Allocation concealment	Blinding of participants, personnel and outcome	Exclusion criteria and refusals number reported	Incomplete outcome data	Outcome and reason why not included in meta-analysis
Borderline personality disorder								
Koons et al. (2001)	20 female veterans with Borderline Personality Disorder.	Weekly DBT skills training groups and 1–1 sessions (*N*=10) vs. TAU and 1–1 therapy (*N*=10) for 6/12.	Potential bias	Potential bias	Potential bias	Adequately described	Potential bias	Both groups reported significant decreases in depressive symptoms and in the number of BPD criteria behaviour patterns, but no decrease in anxiety. There was a reduction in reported intentional self-harm (including suicide attempts) from 50% pre-treatment to 10% at post-treatment in DBT and 20% to 30% in treatment as usual. There was a trend difference in the reduction of para-suicide acts (*z*=1.50, *p*=0.07, one tailed). The only study into BPD—therefore not included in the review.
Depression								
Ruskin et al. (2004)	119 veterans with depression within remote primary care settings.	Participants received either eight face to face sessions with a psychiatrist vs. via telepsychiatry other 6 months.	Adequately described	Potential bias	Potential bias	Potential bias	Adequately described	Both groups improved over the treatment period, with no differences between treatment groups. Participants in both groups were equally adherent to appointments and medication use. There was no between group differences in dropout rates or ratings of satisfaction with treatment. Telepyschiatry was more expensive per treatment session, but disappeared after the psychiatrist travelled more than 22 miles. The only study that utilised telepsychiatry therefore not included in the review.
Gulf War illness								
Donta et al. (2003)	1092 Gulf War veterans with at least 2 of 3 symptom types (fatigue, pain and cognitive) for more than 6 months and at the time of screening.	Twelve, weekly 60–90 min sessions. The interventions were: a) usual care (*N*=271), b) CBT plus usual care (*N*=286); c) exercise plus usual care (*N*=269); or d) CBT plus exercise plus usual care (*N*=266).	Adequately described	Adequately described	Adequately described	Adequately described	Adequately described	The results show improvement in physical functioning at 1 year was 11.5% for usual care, 11.7% for exercise alone, 18.4% for CBT plus exercise and 18.5% for CBT alone. Adjusted OR for improvement in exercise, CBT and exercise plus CBT vs. usual care were 1.07 (95% CI 0.63, 1.82), 1.72 (95% CI 0.91, 3.23) and 1.84 (95% CI 0.95, 3.55), respectively. The only study that investigated Gulf War illness therefore not included in the review.
Panic disorder								
Teng et al. (2008)	35 Veterans with Panic Disorder and co-morbid PTSD.	Ten individual weekly one hour sessions of panic control treatment (*N*=18) vs. psycho-educational supportive therapy (*N*=17).	Potential bias	Potential bias	Potential bias	Adequately described	Potential bias	The PCT group showed significant improvement in panic severity at post-treatment (*N*=18, SMD= −0.87, 95% CI −1.57, −0.17) and panic fear at 3-months follow-up. At 3 months follow-up 63% of participants in the PCT were panic free compared with 19% in the PE-SUP group. There were no changes in general anxiety, depression and PTSD symptoms in either group. The only study that investigated Panic Disorder and co-morbid PTSD therefore not included in the review.
Post-traumatic stress disorder								
Bormann et al. (2008)	33 Korean, Vietnam and Gulf War (1990/1991) with PTSD.	Six weekly group, 90 min sessions of a mantra intervention (*N*=14) vs. delayed treatment control group (*N*=15).	Potential bias	Potential bias	Adequately described	Potential bias	Potential bias	Eighty-eight percent (*N*=29 of the 33) of participants enrolled completed the 6-week intervention (*N*=29). A large effect size, for reducing PTSD symptom severity (*d*= −0.72), psychological distress (*d*= −0.73), and increasing quality of life (*d*=0.70) was reported. This was a pilot study and therefore not included within the review.
Beidel et al. (2011)	35 male Veterans (34 Vietnam and First Gulf War) with combat PTSD.	14 sessions of individual Prolonged Exposure (PE) then group Psycho-education and peer support vs. 14 sessions Trauma Management Therapy (TMT) of PE then group Social Emotional Rehabilitation (SER)	Adequately described	Potential bias	Potential bias	Adequately described	Adequately described	Both groups demonstrated statistically significant reductions in PTSD, but no between group differences on CAPS Total score (*F* (*df*=1,28) =34.08, *p*<0.001), and PCL-M (*F* (*df*=1,28) =6.72, *p*<.01). TMT participants had increased frequency and time engaged in social activities [*p*<0.05]. This study compared the same trauma-focused intervention (PE) with the addition of an emotional rehabilitation in the experimental group and was therefore not included within the review.
Chemtob et al. (1997)	15 Vietnam veterans with PTSD and severe anger.	Twelve 60 min sessions of anger treatment (*N*=8) vs. routine clinical care (*N*=7).	Potential bias	Potential bias	Adequately described	Adequately described	Adequately described	At 18 months there was no significant difference between the two conditions (*N*=15, WMD= −8.59, 95% CI −19.82, 2.64). This was the only study to include veterans with PTSD and co-morbid severe anger and therefore was not included within the review.
Cook et al. (2010)	124 male Vietnam veterans with severe chronic PTSD.	Six 90 min weekly group sessions of Imagery rehearsal (*N*=61) sleep and nightmare management (*N*=63).	Adequately described	Adequately described	Adequately described	Adequately described	Potential bias	There was pre-post change in overall sleep quality and PTSD symptoms for both groups, but not in nightmare frequency (*M*=−0.21, 95% CI −0.63, −0.22). There was *ns* treatment effects for Pittsburgh Sleep Quality Index, Wald test χ^2^ (3) = 1.89, *ns*, weekly number of nightmares, Wald test: χ^2^ (3) =1.23, *ns*, weekly nights with nightmares, Wald test: χ^2^ (3) <1, *ns*, or CAPS, Wald test χ^2^ (1) <1, *ns*. This was the only study that included imagery rehearsal for PTSD and therefore was not included within the review.
Dunn et al. (2007)	101 combat veterans with PTSD and depression.	Fourteen weekly 90 min sessions of group self-management therapy vs. active control group.	Adequately described	Adequately described	Adequately described	Adequately described	Adequately described	At post-treatment follow-up there was no significant difference between the two groups (*N*=77, SMD= −3.17, 95% CI −10.04, 3.70). This was the only study to include a group self-management therapy with veterans with PTSD and co-morbid depression and therefore was not included within the review.
Frueh et al. (2007)	38 Vietnam combat veterans with PTSD.	Fourteen weekly 90 min sessions of group telepsychiatry vs. face to face group therapy.	Potential bias	Potential bias	Potential bias	Adequately described	Potential bias	At post-treatment there were significant group differences, favouring the therapist being in the same room vs. telepsychiatry (*N*=21, SMD=11.53, 95% CI −2.35, 20.71). This was the only study to include a group self-management therapy with veterans with PTSD and co-morbid depression and therefore was not included within the review. This was the only study to include a group telepsychiatry therapy with veterans with PTSD and therefore was not included within the review.
Glynn et al. (1999)	42 Vietnam combat veterans with PTSD.	Eighteen twice weekly prolonged exposure (PE) vs. eighteen sessions of twice weekly PE followed by 16 sessions of weekly behavioural family therapy (BFT) vs. waiting list.	Adequately described	Potential bias	Adequately described	Potential bias	Potential bias	PE reduced re-experiencing and hyperarousal symptoms. These reductions were maintained at 6 months follow-up. Adding BFT to PE had no additional impact on PTSD symptoms (ANCOVA *F*(2, 32)= *p*<0.071). There was a large attrition from the PE-BFT group.
Morland et al. (2004)	17 combat veterans with PTSD.	Eight weekly video-conferencing coping skills group vs. face to face coping skills group.	Potential bias	Potential bias	Adequately described	Adequately described	Potential bias	At post treatment 89% of the patients in the video-conferencing intervention remained in the study compared to 50% in the face-to-face. The video-conferencing patients also attended an average of 6.3 sessions compared to 4.9 sessions in the face-to-face group. Patients reported being satisfied with their particular group treatment and retention of information was also similar for both groups. This was the only study to include a group using coping skills with veterans with PTSD and therefore was not included within the review.
Morland et al. (2010)	125 male combat veterans with chronic PTSD and anger control difficulties. 75% had served in Vietnam.	Anger management therapy delivered in a group setting with therapist in the same room (*N*=64) vs. same treatment via videotele-conferencing (*N*=61).	Adequately described	Adequately described	Adequately described	Adequately described	Potential bias	Participants in both groups showed significant and clinically meaningful reductions in anger symptoms, with post-treatment, 3 and 6 months post-treatment with effect sizes ranging from .12 to .63. Participants in videotele-conferencing demonstrated a reduction in anger symptoms similar to the usual treatment. This was the only study to include a group anger management treatment via teleconferencing with veterans with PTSD and anger control difficulties and therefore was not included within the review.
Price et al. (2007)	14 female veterans with PTSD and chronic pain who were taking prescription analgesics.	Eight weekly mindfulness awareness in body-orientated therapy one-to-one sessions (*N*=7) vs. TAU (*N*=7).	Adequately described	Potential bias	Potential bias	Potential bias	Potential bias	Response rate with 100% attending 7 of the 8 sessions, with all completing post intervention assessment. However, only 3 of the 7 patients in the intervention group returned postal follow-up data. Themes suggested that mindfulness body therapy increased tools to manage pain, and relaxation, increased body/mind connection, trust/safety. The authors do not present any statistical data and therefore was not able to be included within the review.
Schnurr et al. (2003)	360 Vietnam combat veterans.	Thirty weekly TF-group vs. present centred group therapy followed by 5 monthly booster sessions.	Adequately described	Potential bias	Adequately described	Adequately described	Adequately described	At post-treatment no overall differences between the groups were found, although there were significant differences compared to baseline on PTSD severity (*N*=325, FMD=−2.03, 95% CI −5.69, 1.63). This was the only study to include a group TF therapy with veterans with PTSD and co-morbid depression and therefore was not included within the review.
Schnurr et al. (2007)	277 mainly Vietnam combat veterans and 7 active duty personnel.	Ten weekly 90 min PE vs. person-centred therapy.	Adequately described	Adequately described	Adequately described	Adequately described	Adequately described	Participants in the exposure intervention reported a greater reduction of PTSD symptoms compared to the control group (*N*=284, FMD=−7.20, 95% CI −14.15, −0.25). The PE group was more likely to no longer meet criteria for PTSD diagnosis (41% vs. 27.8%; OR, 1.80; 95% CI 1.10, 2.96; *p*=0.1) and achieve total remission (15.2% vs. 6.9%; OR; 2.43; 95% CI 1.10, 5.37; *p* −0.01). This was the only study to include an individual TF therapy with veterans with PTSD and therefore was not included within the review.
Watson et al. (1997)	90 Vietnam veterans with PTSD.	Ten 30 min sessions of relaxation instructions (*N*=30) vs. relaxation instructions plus deep breathing (*N*=30) vs. relaxation instruction, plus deep breathing and thermal biofeedback (*N*=30).	Potential bias	Potential bias	Potential bias	Potential bias	Potential bias	There was limited improvement on only 4 of the 21 PTSD and physiological dependent variables studied. All 21 treatment time interactions were non-significant and no more benefit than being told to relax in a comfortable chair. This was the only study to include anxiety management as the experimental intervention with veterans with PTSD and co-morbid depression and therefore was not included within the review.

**Fig. 2:**
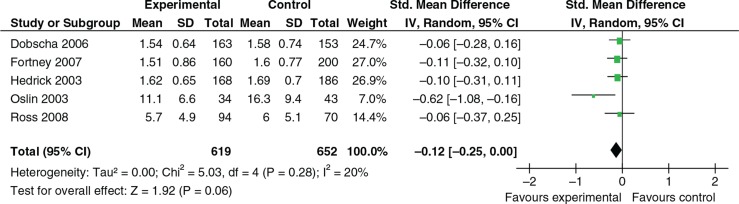
Self-report depression severity.

Psychosocial intervention vs. treatment as usual/waiting list to treat depression Dobscha et al. (2006) investigated depression decision support–collaborative care vs. usual care and Hedrick et al. (2003) collaborative care vs. consult–liaison (usual care). Fortney et al. (2007) tested a collaborative care model within remote isolated clinics via telepsychiatry vs. usual care. Oslin et al., (2003) explored telephone-based disease management in primary care for veterans with depression or at risk drinking vs. usual care. Ross et al. (2008) tested a telephone-based close monitoring program to manage veterans with minor depression in a primary care setting vs. usual care. Figure 2 shows a summary of the outcome effects. There was no significant difference between the collaborative intervention and control in any of the studies or when initial outcomes were combined in meta-analyses (random effects) (*K*=5, *N*=1271; SMD −0.12, 95% CI −0.25, 0.00). There were concerns about methodological quality in all these trials except Dobscha et al. (2006), with three showing concerns on three or more of the validity assessment criteria.

### Psychosocial interventions aimed at reducing insomnia symptoms

**Fig. 3:**
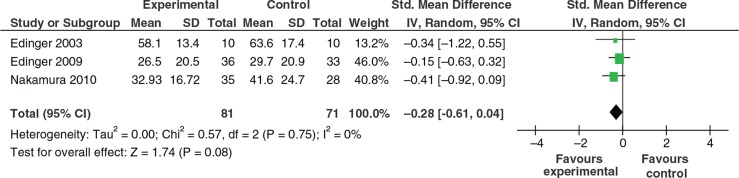
Self-report insomnia clinical measures.

Three studies targeted insomnia (primary and co-morbid) and tested either cognitive behavioural therapy (CBT) or a mindfulness-based intervention against an active control. Edinger and Sampson (2003) used a brief (two sessions) CBT-based intervention vs. a sleep hygiene control. Edinger et al. (2009) provided four sessions of CBT vs. sleep hygiene. Nakamura, Lipschitz, Landward, Kuhn, and West (2010) compared two sessions of weekly mind–body bridging vs. an active sleep education control. There was no significant difference between the experimental intervention and control in any of the studies or when initial outcomes were combined in meta-analyses (random effects) (*K*=3, *N*=152; SMD −0.28, 95% CI −0.61, 0.04), see Fig. 3. There were methodological concerns with all three trials with Edinger and Sampson (2003) and Edinger et al. (2009) failing to meet the standard on at least three of the five validity criteria.

### Trauma-focused psychosocial interventions vs. usual care or waiting list aimed at reducing PTSD symptoms

**Fig. 4:**
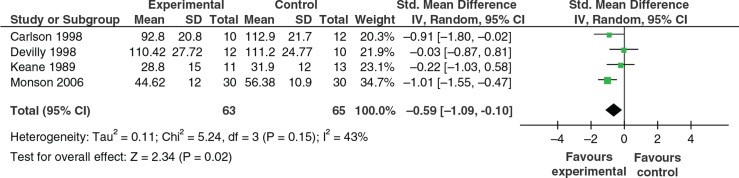
Self-reported PTSD symptom severity.

Self-report data was available from four studies. Carlson et al. (1998) and Devilly et al. (1998) compared EMDR vs. routine clinical care. Keane et al. (1989) compared flooding group techniques vs. wait list control. Monson et al. (2006) tested cognitive processing therapy vs. wait list control. Figure 4 shows a summary of the outcome effects. At initial follow-up, a difference in favour of intervention was apparent when the outcomes of these studies were combined in meta-analyses (random effects) (*K*=4, *N*=128; SMD −0.59, 95% CI −1.09, −0.10). Validity assessment showed that there were methodological concerns with each of these studies (see Table 1), in particular with Carlson et al. (1998), Devilly et al. (1998) and Keane et al. (1989). Monson et al. (2006) was more robustly controlled and achieved the largest effect size.

## Discussion

To our knowledge, this is the first systematic review of psychosocial interventions for veterans presenting with various mental health problems. Twenty-nine RCTs were identified of which 12 were included in meta-analyses. Study quality was variable with significant concerns about methodology in over half of the identified studies, including many entered into meta-analysis. This needs to be taken into account when evaluating the results. There was some evidence for the efficacy of several different trauma-focused psychological therapies delivered on an individual or group basis to treat chronic PTSD with two well-powered and methodologically robust studies reporting particularly positive findings (Monson et al., 2006; Schnurr et al., 2007). There was a lack of evidence to determine the efficacy of trauma-focused therapy delivered by telepsychiatry and of psychosocial interventions for veterans with insomnia. There is evidence from single RCT's for the efficacy of other interventions: dialectical behaviour therapy for female veterans with borderline personality; telephone disease management for depression and at risk drinking; CBT or CBT plus exercise for Gulf War illness and CBT for panic disorder (see Table 2). There is, therefore, limited evidence specific to military veterans on which to base firm recommendations.

We were unable to locate any trials of individual psychosocial interventions such as CBT or interpersonal therapy being evaluated in veteran populations with depression or other anxiety disorders. There are several potential reasons for this that could be usefully explored in future research (Fossey, 2010).

Not surprisingly, there was significant clinical and statistical heterogeneity in the included studies. Seventeen of the trials attempted to reduce traumatic stress symptoms, although the nature of the interventions was diverse. In addition, the total number of hours of the interventions provided across studies varied from two to fifty two hours making results from meta-analyses less meaningful and difficult to generalise from. The results should therefore be interpreted cautiously, although it is noteworthy that a meta-analyses limited to the clinically more homogeneous trauma-focused interventions was positive.

A variety of clinical presentations were included and there were also differences with regards to service history, combat exposure, and time of actual service. These issues may have resulted in differences in the way veterans presented, the duration of their symptoms, and their likelihood to respond to treatment. For example, Korean/Vietnam veterans may be more likely to present with complex, multi-factorial problems and be more difficult to treat than veterans of more recent conflicts or civilians (Beidel et al., 2011). These factors may explain in part the poor outcomes and increase the risk for potential bias (Monson et al., 2006).

The absence of effect of the treatments for depressed veterans is surprising given the evidence for effective psychosocial therapies within primary care for civilians (Butler, Chapman, Forman, & Beck, 2006). This finding may be due in part to civilian therapies not being directly transferable to the veteran population or that other factors such as secondary gain confound results reported in veteran studies (Mossman, 1996). However, it is also arguable that the efficacy of many standard psychosocial interventions has not been adequately evaluated in veteran populations.

## Strengths and limitations

This analysis used a structured and systematic approach based on the Cochrane Collaboration guidance (Higgins & Green, 2009). Three of the authors independently rated the methodological quality of each study. Unfortunately, it was only possible to perform meta-analyses of RCTs for depression, insomnia, and chronic PTSD. Only four studies (Beidel et al., 2011; Donta et al., 2003; Monson et al., 2006; Schnurr et al., 2007) provided any information about whether or not any participants experienced side-effects which might have been attributable to their intervention. The dropout rates were no higher in the intervention than the control groups across studies reviewed, however, suggesting that the interventions did not cause major adverse effects. The absence of a tolerability assessment has been noted as a shortcoming in other psychological treatment reviews (Bisson et al., 2007).

The review was limited to studies published in English, which may have meant that a number of relevant studies were excluded, although we did not identify any abstracts for studies that may have been relevant to the review when we conducted our search. Publication bias is always a concern when conducting a systematic review. However, because of the small number of studies included in meta-analyses it was not possible to explore this in this study. Several of the studies not included in the meta-analysis were of a very high quality and should be considered good examples for future researchers to follow in terms of methodological rigour (Donta et al., 2003; Schnurr et al., 2007).

## Clinical implications

There is enough evidence for clinical services to treat veterans in a way that is based on the evidence base for certain conditions. The results of this review suggest that veterans respond to out-patient trauma-focused psychosocial interventions for chronic PTSD on a one-to-one or group basis with the therapist within the same room. This is consistent with the evidence from meta-analyses of civilian studies (Bisson et al., 2007) and supports a recommendation that trauma-focused interventions should be offered to all veterans with chronic PTSD.

There is also some evidence for dialectical behaviour therapy for treating borderline personality disorder in female veterans, telephone disease management for depression and at risk alcohol abuse, CBT and exercise for Gulf War illness, and CBT or panic control treatment for panic disorder co-morbid with PTSD, but replication is required.

The lack of efficacy of collaborative psychosocial interventions for veterans with depression suggests that, at present, it is appropriate to offer veterans alternative treatments for depression that have been shown to be effective in civilian populations. Caution should, however be exercised until efficacy studies of treatments such as CBT and interpersonal psychological therapy have been conducted on veterans with depression.

Veterans with mental health problems remain a difficult group for military and civilian mental health services to engage in mental health treatment programmes (Improving Access to Psychological Therapies [IAPT], 2009). Interventions may be more effective for veterans if delivered in a timely fashion post discharge from military service. It would be helpful for future research to consider how best to improve access and uptake of interventions by veterans separated from service at an earlier stage.

## Implications for future research

Further well-designed RCTs of existing civilian and veteran-specific psychosocial interventions for common mental disorders in veteran populations are required. There is a need for further comparison studies of active psychosocial treatments and the role, if any, of pharmacological treatments in combination with psychosocial therapy. The role of technology that utilises psychosocial therapy in novel formats, for example, manuals, telepsychiatry (video-conferencing), telephone, and website-based interventions that allow veterans in remote places or who will not enter psychiatric treatment settings due to stigma, and unhelpful beliefs about health providers need to be further developed and thoroughly evaluated.

There is a need for larger effectiveness trials of psychosocial therapies that are conducted in non-military settings and within cultures other than the United States. Further trials should also consider adverse events, tolerability of the treatment provided, carefully controlled for any additional intervention, and evaluate cost effectiveness.
